# Bandwidth Optimization of a Textile PIFA with DGS Using Characteristic Mode Analysis

**DOI:** 10.3390/s21072516

**Published:** 2021-04-04

**Authors:** Bashar Bahaa Qas Elias, Ping Jack Soh, Azremi Abdullah Al-Hadi, Prayoot Akkaraekthalin, Guy A. E. Vandenbosch

**Affiliations:** 1 Advanced Communication Engineering (ACE) CoE, Faculty of Electronic Engineering Technology, Pauh Putra Campus, Universiti Malaysia Perlis (UniMAP), Arau 02600, Malaysia; azremi@unimap.edu.my; 2 Department of Communication Engineering Techniques, Imam Ja’afar Al-Sadiq University, Baghdad 10052, Iraq; 3 ESAT-WAVECORE Research Division, KU Leuven, Kasteelpark Arenberg 10, 3001 Leuven, Belgium; guy.vandenbosch@esat.kuleuven.be; 4 Department of Electrical and Computer Engineering, Faculty of Engineering, King Mongkut’s University of Technology North Bangkok (KMUTNB), 1518 Pracharat 1 Rd., Wongsawang, Bangsue, Bangkok 10800, Thailand; prayoot.a@eng.kmutnb.ac.th

**Keywords:** planar antennas, characteristic mode analysis, defected ground structure

## Abstract

This work presents the design and optimization of an antenna with defected ground structure (DGS) using characteristic mode analysis (CMA) to enhance bandwidth. This DGS is integrated with a rectangular patch with circular meandered rings (RPCMR) in a wearable format fully using textiles for wireless body area network (WBAN) application. For this integration process, both CMA and the method of moments (MoM) were applied using the same electromagnetic simulation software. This work characterizes and estimates the final shape and dimensions of the DGS using the CMA method, aimed at enhancing antenna bandwidth. The optimization of the dimensions and shape of the DGS is simplified, as the influence of the substrates and excitation is first excluded. This optimizes the required time and resources in the design process, in contrast to the conventional optimization approaches made using full wave “trial and error” simulations on a complete antenna structure. To validate the performance of the antenna on the body, the specific absorption rate is studied. Simulated and measured results indicate that the proposed antenna meets the requirements of wideband on-body operation.

## 1. Introduction

Wearable antennas have recently received considerable attention due to their cost-effectiveness, light weight, flexibility, and ease of integration into clothes [1,2,3,4]. The main materials used are usually based on textiles and various flexible polymers. Generally, textile materials have a higher loss than standard conductors and will reduce total radiation efficiency. Despite that, it remains the most popular material being used to fabricate wearable antennas as conductive textiles can be directly incorporated in clothes [5,6,7,8,9].

Characteristic mode analysis (CMA) is a method used in electromagnetics, which provides insight into the inherent resonant characteristics of a structure by finding and examining the structure’s basic modes [10,11,12,13]. Any physical object has a set of modes dependent on its structure, materials, and boundary conditions. The theory of CMA was first introduced in [14] and later refined by [15,16]. It can be very effectively used in the antenna design process. The presence of the modes is independent of the excitation. However, different types of excitations at various locations can be used to meet different operational requirements.

The defected ground structure (DGS) technique is very commonly used in literature for improving bandwidth while maintaining structural simplicity. It involves the integration of single or multiple slots in the ground plane of planar circuits and antennas. Such integration of DGS with antennas provides structural miniaturization and surface wave suppression, which then reduces the effects of coupling to the human user [17,18,19,20]. Conventionally, in planar microstrip circuits, a DGS is located under a microstrip line, and it perturbs the existing electromagnetic fields. Trapped electric fields give rise to the capacitive effect (C), while the surface currents around the defect cause an inductive effect (L) [21].

This work presents an optimized planar inverted-F antenna (PIFA) structure with DGS integrated on its ground plane. The optimization procedure for both the DGS shape and its location is performed by studying the resonant modes of the ground plane using the CMA approach. This consequently results in an optimized method to identify the bandwidth increase for the proposed PIFA with DGS. This approach also makes the normal resource- and time-consuming antenna optimization procedure to be more efficient. The radiator of the PIFA is designed based on a unique compact rectangular patch and is integrated with circular meandered rings.

This paper is organized as follows. The structure of the proposed antenna is first described in Section 2, followed by the simulated performance of the antenna in Section 3. The on-body evaluation of the antenna is then discussed in Section 4, followed by the measurement results and analysis in Section 5. Finally, Section 6 concludes this work.

## 2. Antenna Design

The structure of the antenna is initially based on [22]. The antenna proposed in this reference initiates the use of the chassis as part of the radiator. This increases the effective aperture of the antenna, thus enabling a more compact antenna, which is especially important for operation at lower frequencies. The structure in [22] consists of five layers:

- a 50 × 25 mm^2^ ground plane made using a 0.17 mm thick Shieldlt conductive textile (with conductivity, σ = 1.18 × 105 S/m),
- a 3 mm thick felt substrate (*εr* = 1.3, tan*δ* = 0.044),
- a 5 mm air gap,
- a 1.6 mm thick FR4 substrate (*εr* = 4.3, tan*δ* = 0.044),
- and a patch built using a 0.17 mm thick copper conductor.

**Figure 1:**
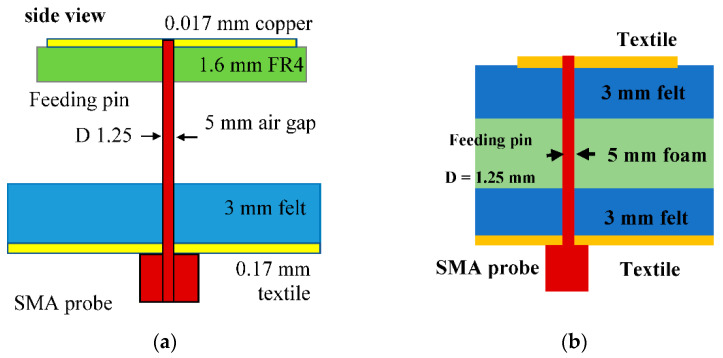
Side view of the antenna (**a**) from the previous reference [22] (© 2019 IEEE), (**b**) proposed antenna.

This structure is shown in Figure 1a. To improve flexibility for this antenna, the rigid FR4 substrate (layer (iv)) in the previous work is replaced with a 3 mm thick felt in this proposed work, whereas the copper conductor in layer (v) is replaced with the 0.17 mm thick Shieldlt textile. To ensure a practically realizable structure, the air gap in layer (iii) from the previous work is replaced by a foam substrate (*εr* = 1.06), as shown in Figure 1b.

**Figure 2:**
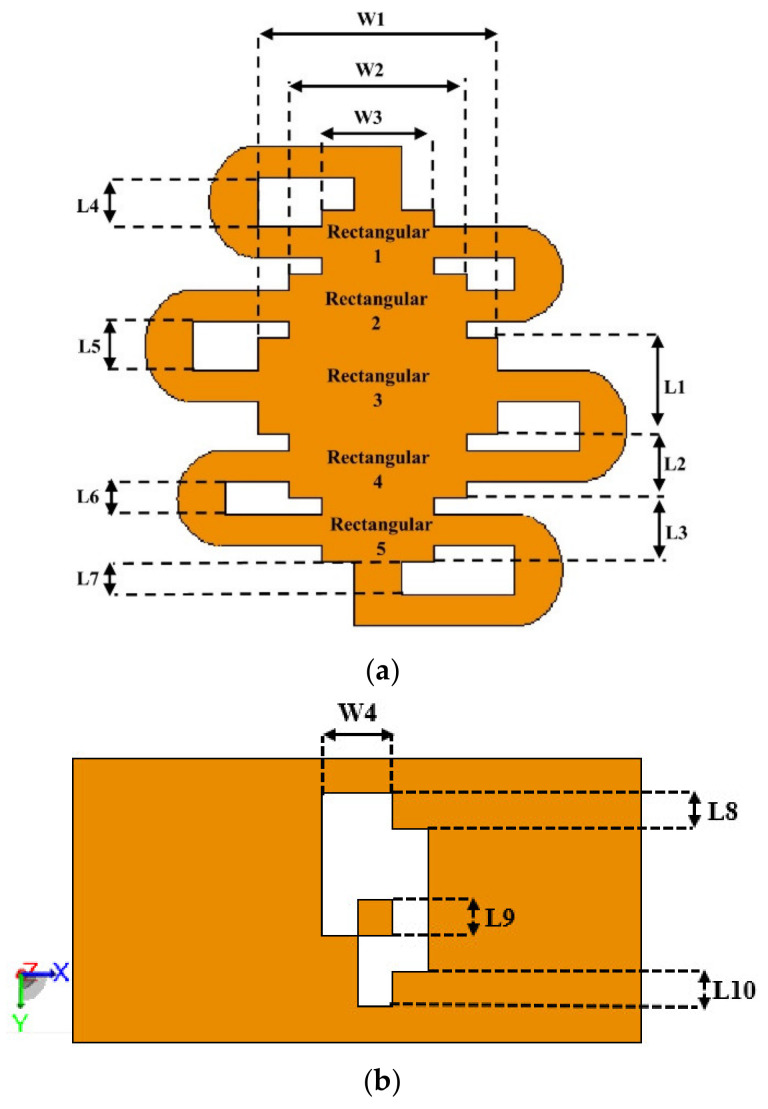
(**a**) RPCMR structure, (**b**) DGS structure.

The main radiating structure for the proposed antenna is located on the top layer in the form of a rectangular patch. It is formed using Shieldlt conductive textile. Circular meandered patches are then connected with the patch, as shown in Figure 2a. Similar to the previous design, the ground and lower substrate layer are also made fully using textiles. A 5 mm thick foam layer separates the two felt substrates. The aim of using multilayered flexible substrates is to enhance the antenna’s narrow bandwidth with an increased substrate thickness. Despite enhancing the profile of the antenna, the overall thickness can still be practically integrated into winter apparel or even building insulation to provide sufficient warmth to users, while providing communication functionality due to the material’s flexibility and lightness [23,24]. A standard 50 Ω SMA connector is used to excite the antenna coaxially from beneath the ground plane. Details of the optimized parameters are summarized in Table 1.

## 3. Optimization Procedure and Results

### 3.1. DGS Design and Optimization

The basic concept of DGS is based on a so-called photonic bandgap (PBG) structure, with “defects” that are etched in the ground plane. The electromagnetic field can be controlled by using these defects, enabling circuits to become more compact [25].

**Figure 3:**
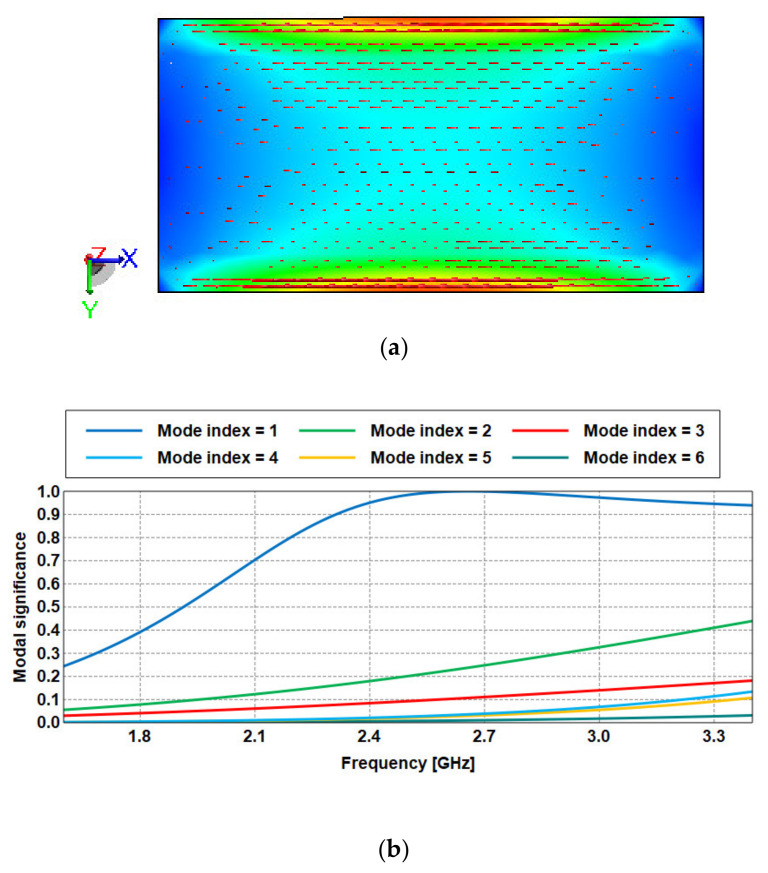
(**a**) Current distribution in the ground plane, (**b**) modal significance.

Initially, the proposed ground plane in Figure 3a dimensioned at 50 × 25 mm^2^ (0.35 × 0.17 λg^2^) is analyzed based on CMA to study the efficiency of the generated modes and to identify the dominant ones. It is known that defects in the ground plane alter the current distribution, and this feature is used to change the structure’s electromagnetic characteristics. Thus, by including slots in the ground plane, additional resistances, capacitances, and inductances can be generated in any planar structure. From Figure 3b, it is found that only the first mode is active, as indicated by its close-to-unity modal significance. The other modes (shown from 2 to 6) are less important.

**Figure 4:**
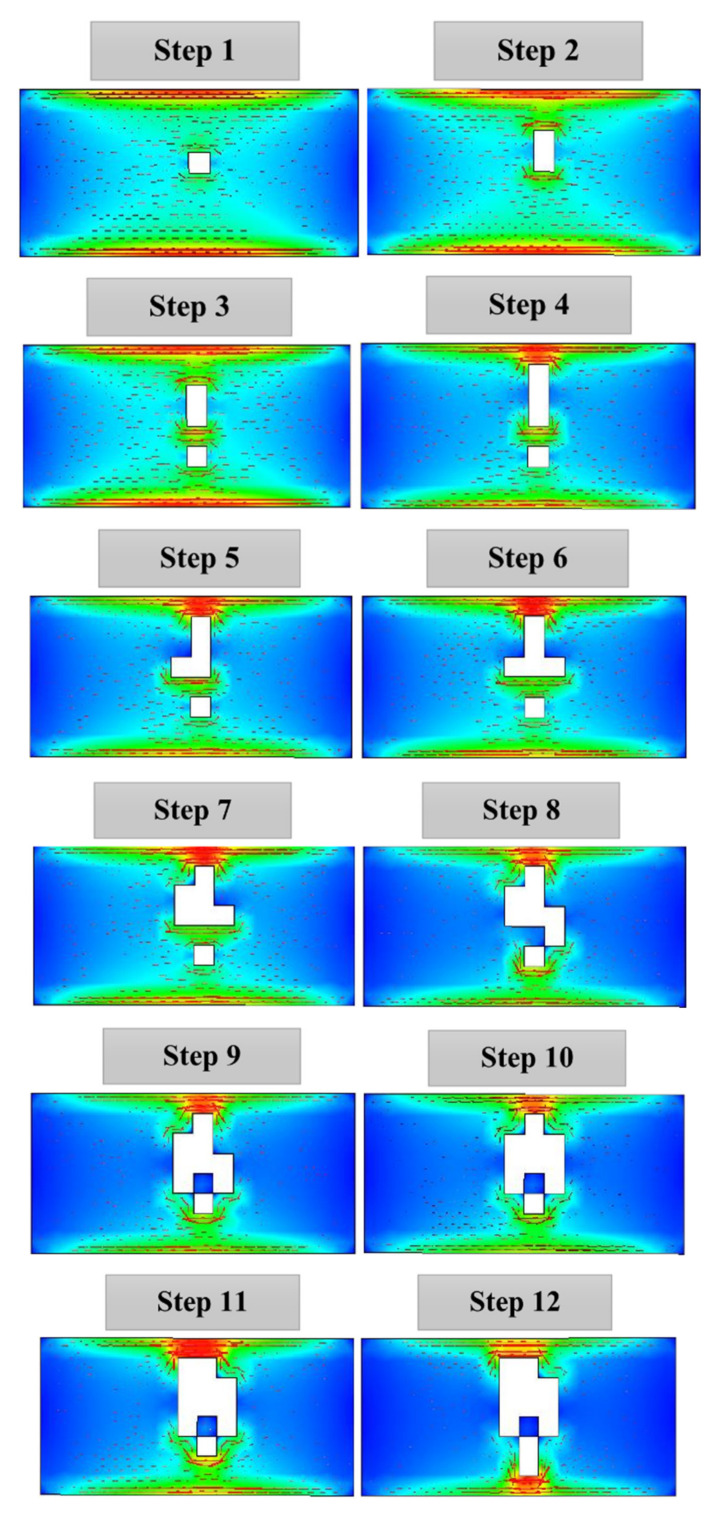

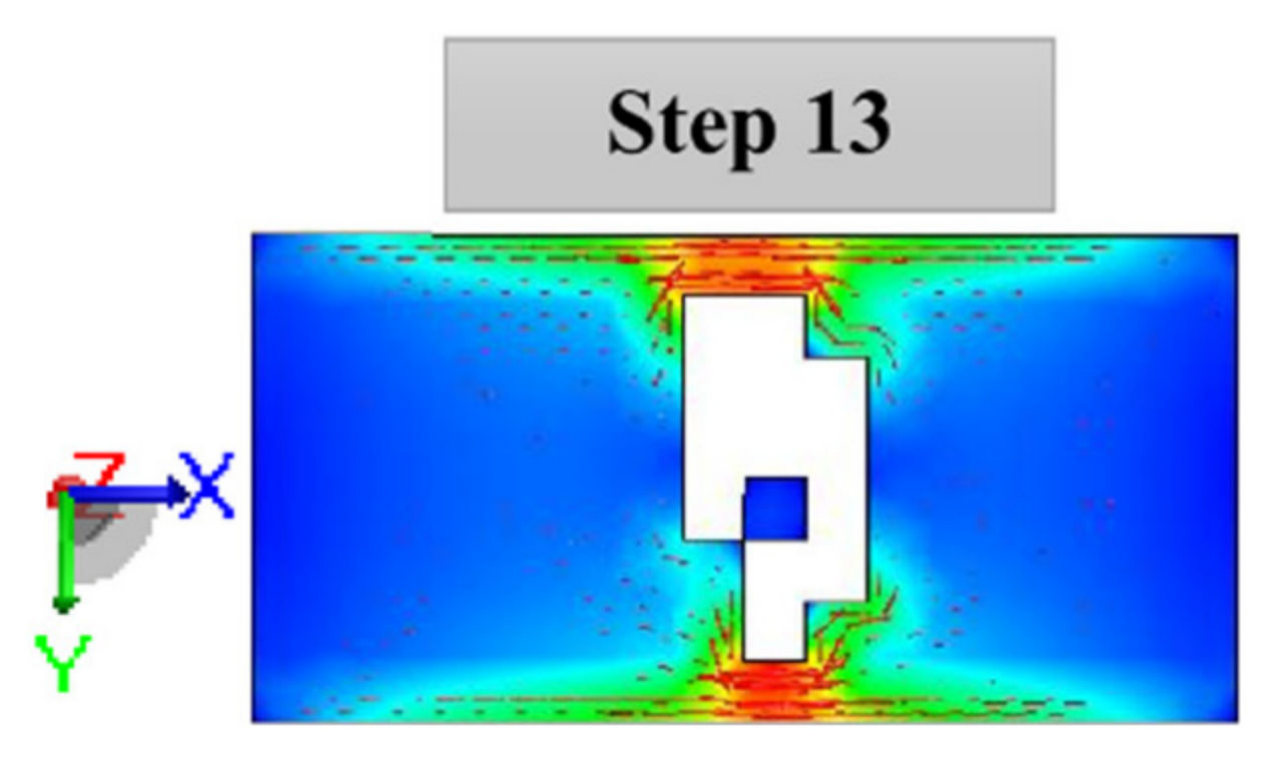
Surface currents in the ground plane in each step of adding slots.

**Figure 5:**
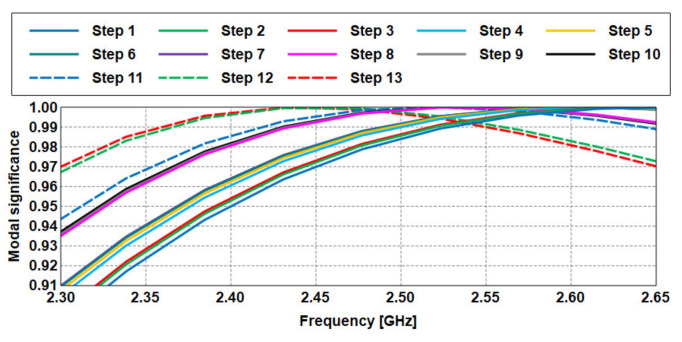
Modal significance of each step of slot addition.

The next step involves shifting this main resonant mode towards the target frequency of 2.45 GHz to ensure efficiency when this structure is being integrated with the rest of the antenna layers. The DGS structure is formed and studied gradually in several steps, as depicted in Figure 4. It is observed that the surface currents are concentrated on the upper and lower edges of the ground plane, and that high-intensity areas are created at the middle of the slot edges as the number of slots increases. At the same time, the resonant frequency of the mode decreases with the addition of slots, as shown in Figure 5, until the target 2.45 GHz frequency is achieved. This is then set as the final DGS structure.

The different design steps for the DGS from the ground plane in this work can be summarized as follows:

1. First, the PEC ground plane is analyzed alone without the rest of the antenna parts.
2. Next, the modal significance result based on the dimensions of the ground plane is assessed to identify the dominant modes and their frequencies using CMA.
3. The eigen currents are then generated on the surface of the ground plane using CMA to identify the areas having the lowest values of currents. The etching of the DGS is initialized at these locations.
4. Finally, slots are then gradually inserted until the desired resonant frequency is achieved. This is observed from the modal significance curves being significant (>0.7) at the desired operating frequency. This enables the estimation of the final structure of the DGS.

Slot sizes are carefully chosen and increased gradually to avoid significant shifting in operating frequency from each step. The slot structure is not limited to a rectangular shape, and it is possible to use other geometries as well to result in DGS with different final geometries but same modal significance response.

### 3.2. Patch Structure

A conventional rectangular patch for operation at 2.45 GHz in the given layer structure has a size in the order of 50 × 50 mm^2^ [26]. Since this size is unsuitably large, our design aims at reducing these dimensions by using a diamond-shaped patch in combination with meandered rings on the two sides, yielding an RPCMR: rhombic patch with curved meandered rings.

**Figure 6:**
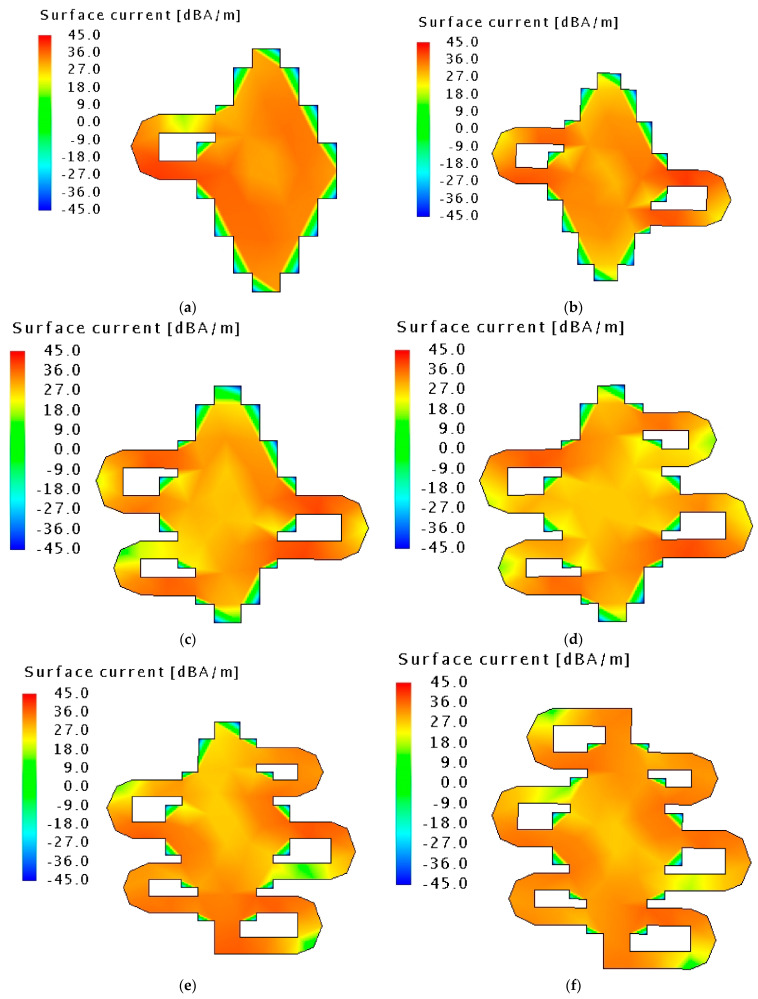
Surface current distribution of the fundamental CMA mode on the patch. In each step an additional meandered ring is added, as follows: (**a**) with one ring (**b**) with two rings (**c**) with three rings (**d**) with four rings (**e**) with five rings (**f**) with six rings.

**Figure 7:**
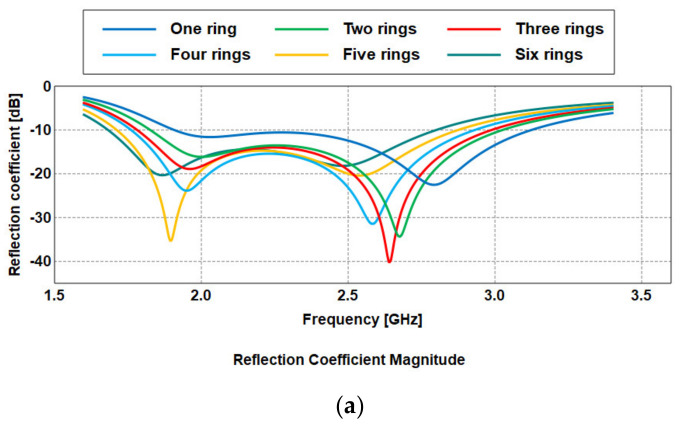

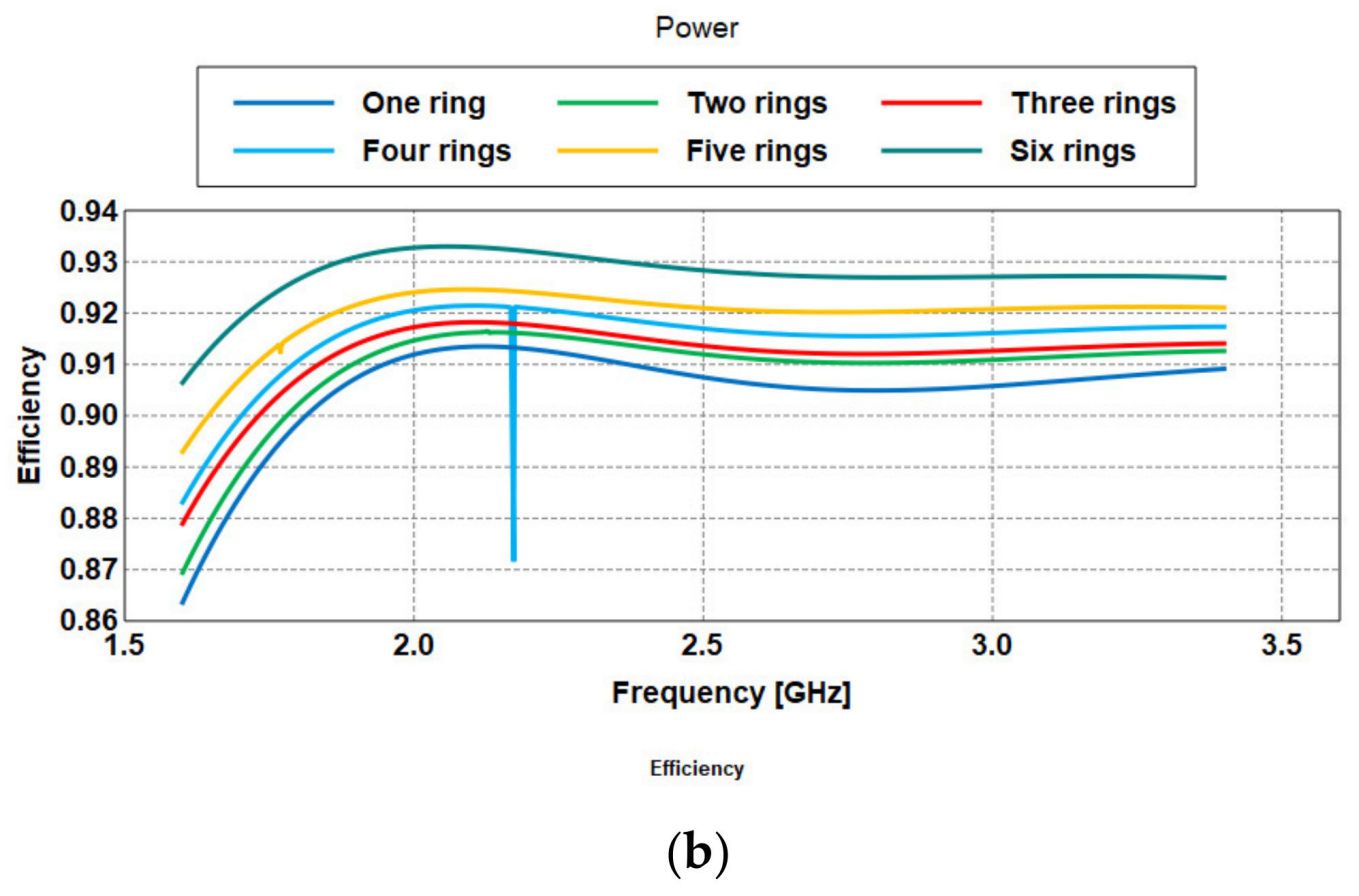
Simulation results from each design step: (**a**) reflection coefficient, (**b**) efficiency.

Meandered structure is a popular technique to improve compactness. It creates longer electrical current paths inside a relatively small area. When more rings are gradually added, a more uniform current distribution throughout the overall structure is observed. After considerable computational efforts, this study resulted in an antenna with six meandered rings with a size reduction of approximately 64% compared to a conventional patch. The current distribution in each of the design steps of the RPCMR is presented in Figure 6a–f. As seen in Figure 7a, the operating frequency bands for the different steps are: 1.91–3.11 GHz in step 1, 1.82–3.02 GHz in step 2, 1.78–2.98 GHz in step 3, 1.76–2.93 GHz in step 4, 1.71–2.87 GHz in step 5, and 1.68–2.8 GHz in step 6. The radiation efficiency is improved with increasing number of rings to 93% at 2.45 GHz, as shown in Figure 7b.

### 3.3. Optimal Ground Location

**Figure 8:**
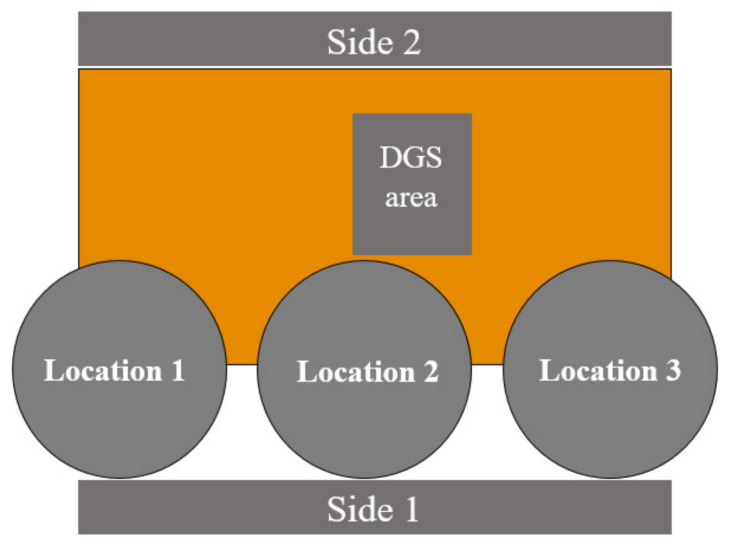
Different locations on the ground plane.

After establishing the patch shape of the RPCMR, its location with respect to the ground needs to be optimized; see Figure 8.

**Figure 9:**
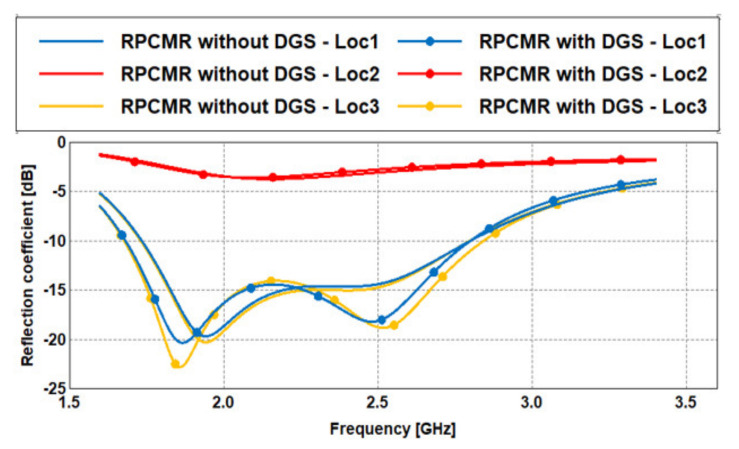
Reflection coefficients of the complete RPCMR antenna including substrates and feeding (at different locations).

A thorough study was performed, pointing out that positioning the RPCMR in a corner yields the largest bandwidth. The optimum there was found based on a dimensional resolution of 0.1 mm, both in *x*- and *y*-direction. The reflection coefficients for two different corner locations, left at (−19.92 mm, −12.11 mm) and right at (19.92 mm, −12.11 mm) and the location in the center, and for grounds without and with DGS, are shown in Figure 9. For the corner locations, a dual-band behavior is obtained with a large −10 dB bandwidth. For the center location, the reflection coefficient is totally unsatisfactory. Note that the coordinates are the coordinates of the center point of the patch with respect to the center of the ground. The results from placing the RPCMR on the left and right sides are not perfectly symmetrical because the antenna itself is not mirrored. It is clearly seen that the DGS technique can further improve the antenna bandwidth.

**Figure 10:**
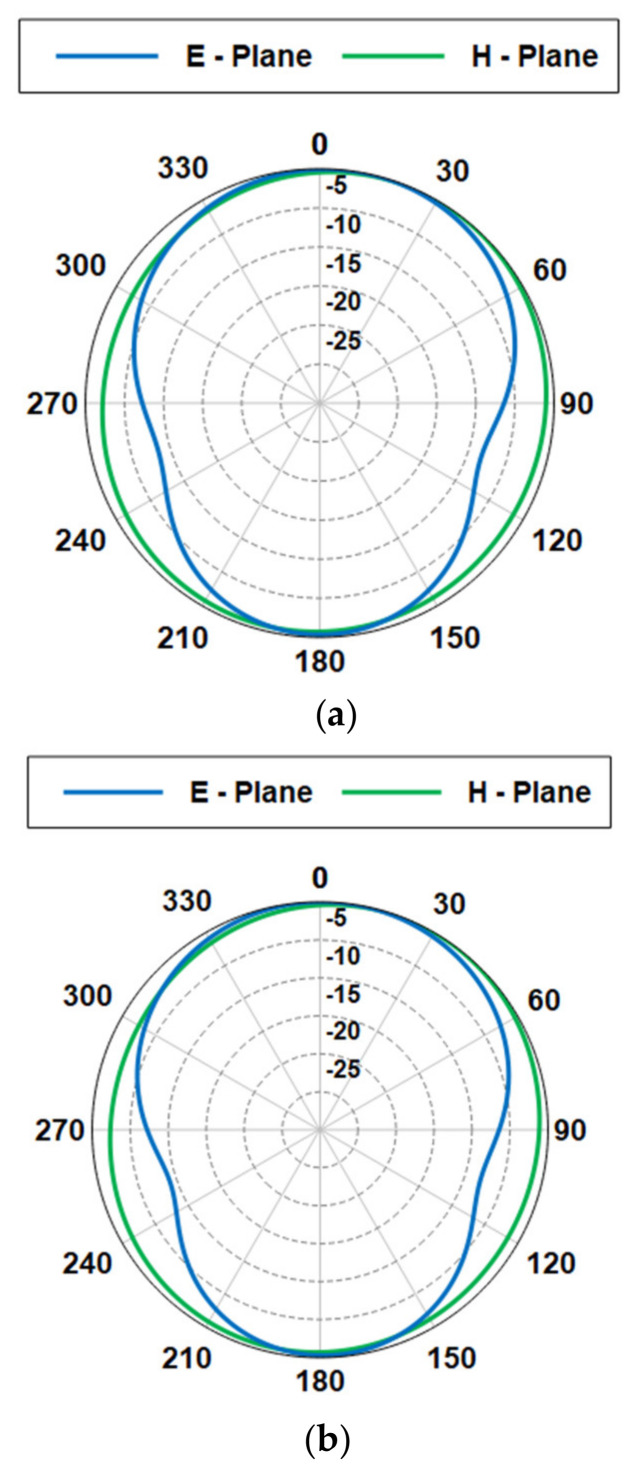
Normalized radiation patterns of the proposed antenna at 2.45 GHz (E- and H-plane cuts): (**a**) without DGS, (**b**) with DGS.

**Figure 11:**
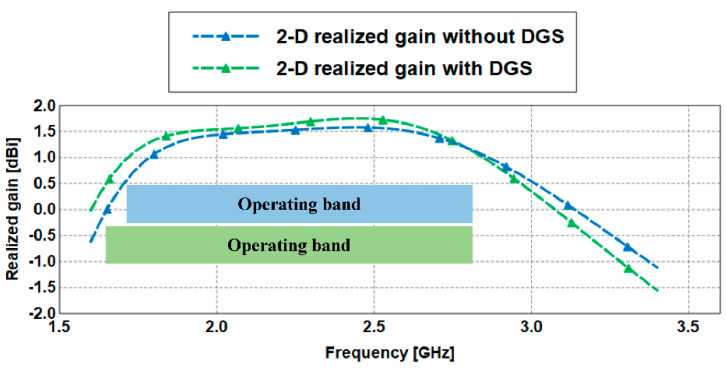
Realized gain of the RPCMR antenna with and without DGS.

The antenna radiation patterns in the E- and H-planes are presented in Figure 10. The antennas with and without DGS qualitatively produce the same radiation patterns. The realized gain is depicted in Figure 11. It is observed that the DGS technique slightly increases the gain in the operating band. The bandwidth and radiation efficiency at each location are summarized in Table 2.

## 4. Antenna on Body Evaluation

**Figure 12:**
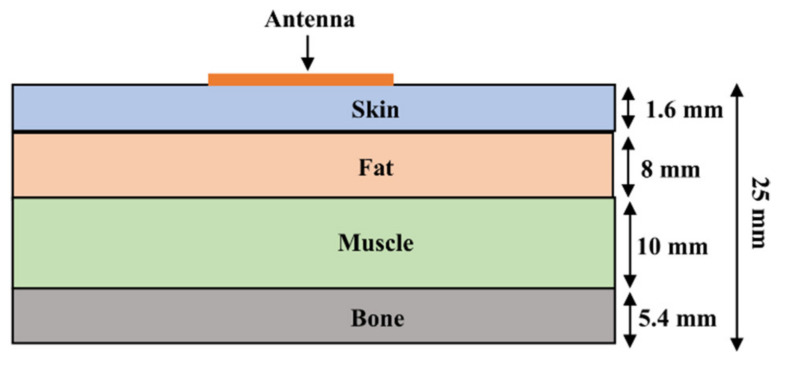
Side view of the heterogeneous model.

In this work, a heterogeneous model is used to represent the human body. It consists of four layers of tissue-skin, fat, muscle, and bone, as shown in Figure 12 [27,28,29].

**Figure 13:**
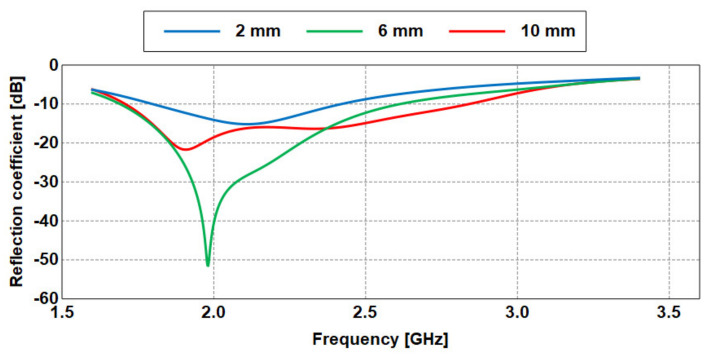
Simulated reflection coefficient of the antenna placed on body with different spacings.

To assess the antenna performance when placed on-body (OB), the antenna is placed at distances of 10 mm, 6 mm, and 2 mm from the human model. Such spacing mimics the natural spacing between the antenna and body due to clothing. The reflection coefficients when placed on-body shown in Figure 13 indicate that the antenna is operational across a much wider frequency band. For instance, the antenna operates from 1.706 GHz to 2.844 GHz (1.138 GHz of bandwidth) when placed at 10 mm.

This 10 dB impedance bandwidth is significantly decreased to 0.92 GHz (from 1.69 GHz to 2.61 GHz) and 0.62 GHz (from 1.79 GHz to 2.41 GHz) when the spacing is reduced to 6 mm and 2 mm, respectively, due to coupling to the human body. Thus, the bandwidth decreases when the distance between the antenna and the heterogeneous model is reduced.

**Figure 14:**
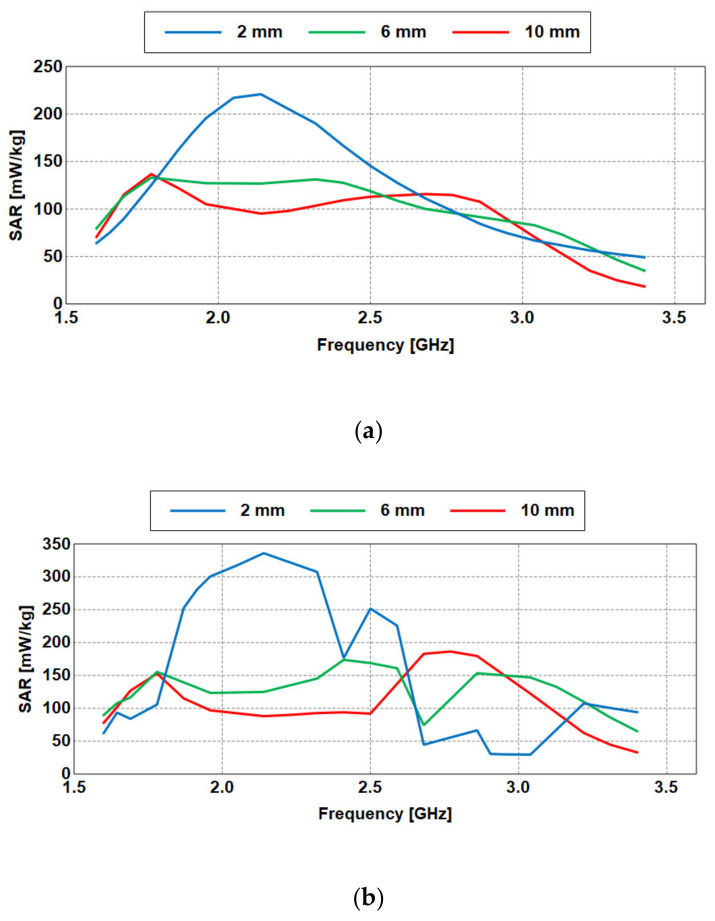
Maximum SAR values for the antenna on body for different distances: (**a**) 1 g, (**b**) 10 g.

The specific absorption rate (SAR) [30] is defined as electromagnetic energy absorbed per unit biological human tissue mass when exposed to a radiating device. SAR values are usually averaged over a certain volume of exposed biological tissue (typically 1 g or 10 g). Figure 14 presents the SAR levels obtained for the antenna placed on the heterogeneous model. Values within the standard limits are obtained as illustrated in Table 3.

**Figure 15:**
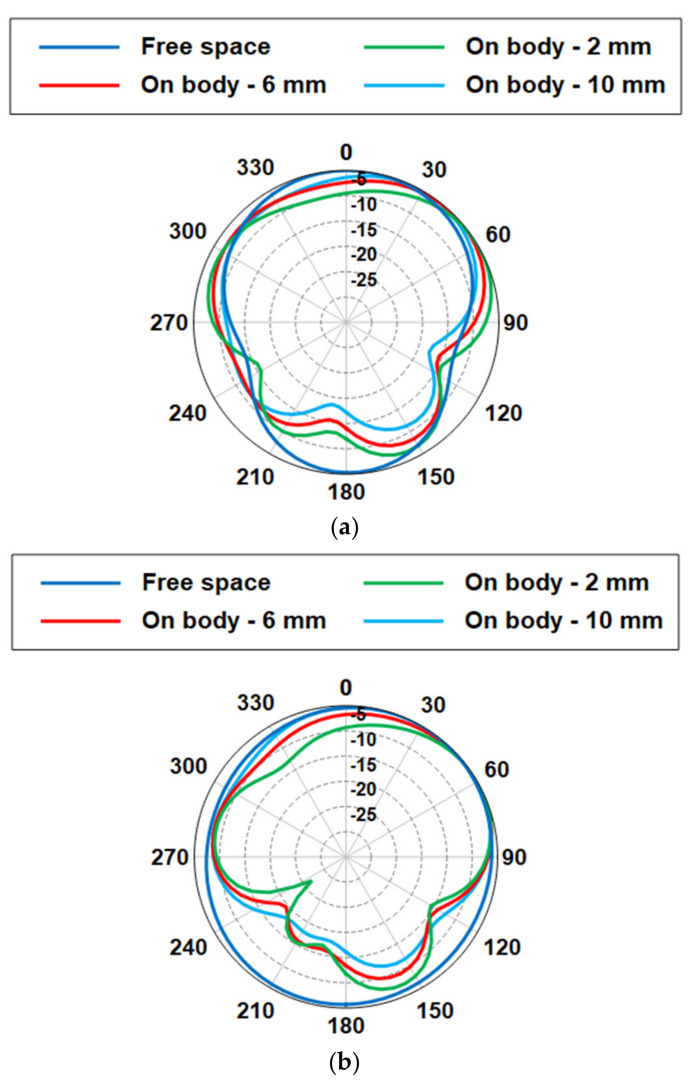
Normalized radiation patterns of the antenna when placed on body for different spacings: (**a**) E-plane cut, (**b**) H-plane cut.

Another aspect when the antenna is placed close to the body is the reduction of the back radiation; see Figure 15. In general, the radiation performance to the front then is improved. In our case, the front-to-back ration (FBR) increases to 10.94 dB at 2.45 GHz for a spacing of 10 mm.

Further, the antenna gain is reduced compared to free space due to the absorption by the human tissue; see Table 4.

## 5. Prototype and Measurements

**Figure 16:**
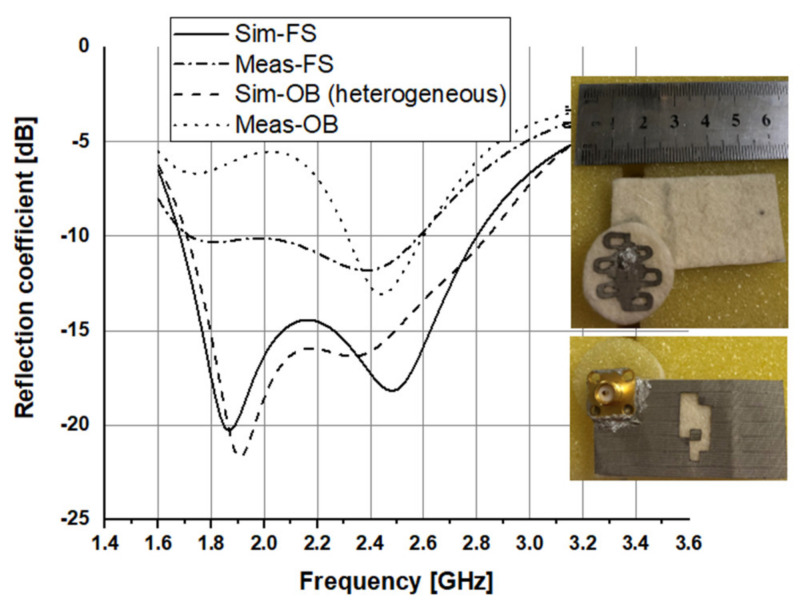
Simulated and measured reflection coefficients at 2.45 GHz.

**Figure 17:**
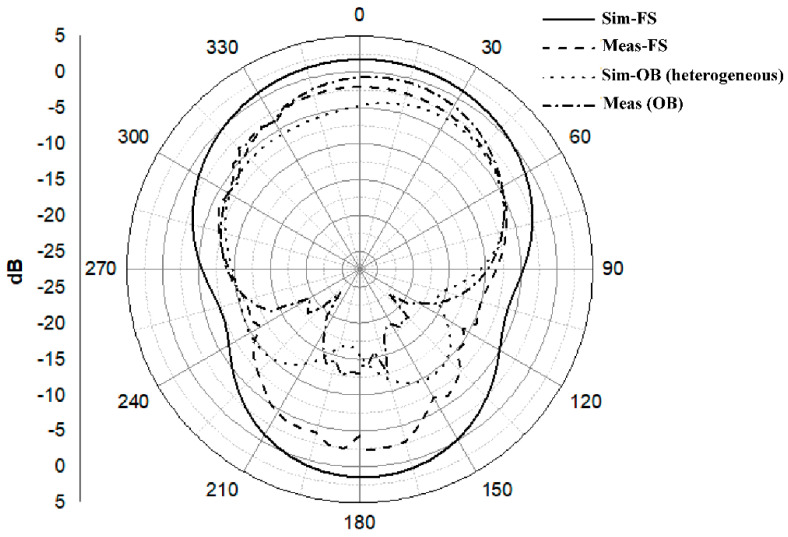
Simulated and measured radiation patterns at 2.45 GHz.

A prototype of the proposed antenna was fabricated, using manual cutting tools, and evaluated experimentally. The measured reflection coefficient in Figure 16 indicates a −10 dB reflection bandwidth of around 860 MHz (from 1.72 to 2.58 GHz). When evaluated on the chest with 10 mm between the antenna and the human body, no huge changes are seen in the upper part of the operating frequency band. However, the lower resonant frequency shifts down and deteriorates. Figure 17 compares the simulated and measured radiation patterns. In general, there is an acceptable agreement between simulated and measured patterns. Table 5 summarizes all results.

**Figure 18:**
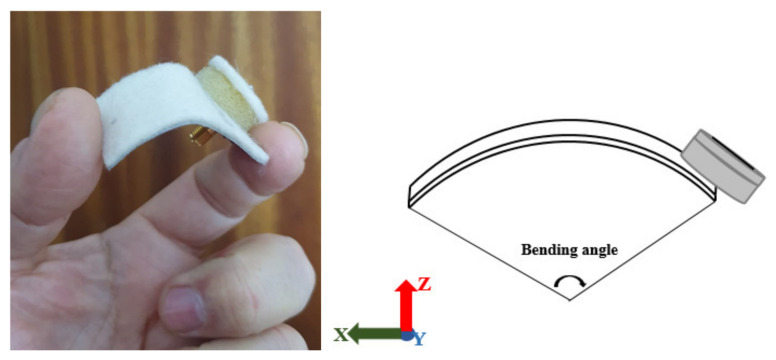
Photograph of the RPCMR antenna when bent.

**Figure 19:**
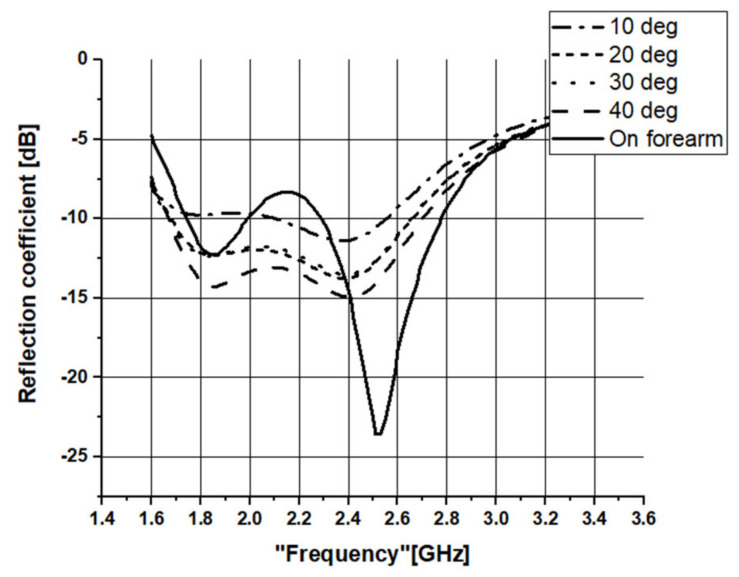
Reflection coefficients of the RPCMR antenna when bent at different angles and when placed on the forearm.

The flexible characteristic of the proposed antennas is further studied by bending the antenna as shown in Figure 18 at different angles (40°, 30°, 20°, and 10° from the least to the worst degree of bending). The measured reflection coefficient when the antenna is bent at 40° is −14.94 dB at 2.41 GHz. An overall bandwidth of 1032 MHz is achieved. Next, by increasing the bending angle to 30° and 20°, the operating frequency of the antenna slightly decreases to 2.4 GHz and 2.39 GHz, with a reflection coefficient of −13.51 dB and −13.77 dB, respectively. The bandwidth produced from these two configurations are 986 MHz and 979 MHz, respectively. Finally, the most extreme bending angle of 10° further shifts the resonant frequency to 2.38 GHz, with −11.38 dB of reflection coefficient and the smallest impedance bandwidth of 448 MHz. The increase in bending (reduction in bending angle) reduces the antenna bandwidth with a slight decrease in the operating frequency. This is due to the variation of the effective length as the bending increases [VIII], and is evident from Table 6. The gradually bent antenna (from the flat case to 40°, 30°, 20°, and 10°) increases the effective length of the RPCMA radiator at the long edge of the chassis (along the y-direction). Besides this, when the proposed antenna is placed at a distance of 5 mm from the forearm, two resonant bands are obtained centered at 1.85 GHz and 2.52 GHz, with a bandwidth of 249 MHz and 487 MHz, respectively. The reflection coefficients for the bending investigation are presented in Figure 19 and summarized in Table 6.

The performance of the proposed antenna is compared with other state-of-the-art wireless body area network (WBAN) antennas designed for operation in the same frequency band in Table 7. The proposed antenna features the largest bandwidth and improved efficiency compared to literature. Moreover, the operating bandwidth is improved compared to other flexible antennas in [22,31,32,33,34,35]. Its compact size of 0.35 × 0.17 λg^2^ also makes the proposed antenna among the smallest available wideband antennas, enabling its easy integration into wearable systems. Finally, the proposed antenna also provides a higher gain compared to [35] and enhanced radiation efficiency relative to the antennas in [22,31,32,33].

## 6. Conclusions

In this paper, a compact dual-band RPCMR antenna for on-body applications was proposed, designed, prototyped, and measured. The characteristic mode analysis was used to optimize the use of a defected ground structure in order to maximize the bandwidth. The method of analysis (CMA) proposed in this study is appropriate to be used in evaluating antennas made using different flexible or nonflexible materials. This is because the initial simulations in the CMA method proposed do not account for the substrate and excitation. Instead, this method only accounts for the metallic parts of the antenna, regardless of whether it is flexible or not. This, then, reduces the required time and effort in the optimization, besides simplifying the design procedure. This resulted in a fractional bandwidth of 49.04%, a gain of 3 dBi, and an average efficiency of 93%. Moreover, the antenna is compact in size (0.35 × 0.17 λg^2^), lightweight, and flexible, which makes it a prospective candidate for WBAN applications.

## Figures and Tables

**Table 1:** Summary of the antenna dimensions.

Parameter	Value (mm)	Parameter	Value (mm)
*W1*	9.3	*L4*	1.86
*W2*	6.82	*L5*	1.86
*W3*	4.34	*L6*	1.24
*W4*	6.25	*L7*	1.24
*L1*	3.72	*L8*	3.125
*L2*	2.48	*L9*	3.125
*L3*	2.48	*L10*	3.125

**Table 2:** Reflection coefficient, realized gain, bandwidth, and efficiency.

Location	Resonant Frequency (GHz)		Reflection Coefficient (dB)		Realized Gain (dB)		Bandwidth (GHz)		Efficiency %	
	Without DGS	With DGS	Without DGS	With DGS	Without DGS	With DGS	Without DGS	With DGS	Without DGS	With DGS
1	1.95	1.87	−19.66	−20.34	2.5	2.5	1.066	1.118	92	93
	2.45	2.45	−14.59	−18.09	4.0	3.0			93	93
2	N/A									
3	1.94	1.86	−20.26	−22.8	2.5	3	1.068	1.166	92	92
	2.4	2.5	−15.03	−18.74	4	3			93	92

**Table 3:** Simulated SAR Values.

Distance (mm)	SAR Values (W/kg)			
	1 g Cube		10 g Cube	
	1.87 GHz	2.45 GHz	1.87 GHz	2.45 GHz
10 mm	0.115	0.093	0.122	0.111
6 mm	0.139	0.172	0.130	0.124
2 mm	0.253	0.210	0.163	0.157

**Table 4:** Simulated realized gain results at *θ* = 0°.

Distance (mm)	Realized Gain (dBi)	
	1.87 GHz	2.45 GHz
Free space	1.46	1.75
10 mm	−1.75	−1.44
6 mm	−3.81	−3.72
2 mm	−7.18	−7.05

**Table 5:** Simulated and measured performance of the proposed antenna.

Results		Resonant Frequency (GHz)	Reflection Coefficient (dB)	Bandwidth %/(MHz)
Sim.	FS	1.87/2.45	−20.34/−18.09	49.04/1118
	OB	1.9	−21.71	49.74/1131
Meas.	FS	1.8/2.38	−10.33/−11.82	40.16/864
	OB	2.44	−13.09	11.44/281

**Table 6:** Experimental results of the RPCMR antenna when bent at different angles and when placed on the forearm.

Case	Operating Frequency (GHz)	Reflection Coefficient (dB)	Bandwidth (MHz)
Bent at 10°	2.38	−11.38	448
Bent at 20°	2.39	−13.77	979
Bent at 30°	2.4	−13.51	986
Bent at 40°	2.41	−14.94	1032
Bent on forearm	1.85	−12.27	249
	2..52	−23.68	487

**Table 7:** Comparison of the proposed antenna with other wideband textile-band antennas available in literature.

Ref	Antenna Dimensions (λg^2^/mm^2^)	Operating Frequency (GHz)	Bandwidth (%)	Gain (dBi)	Flexible?	Efficiency %
[31]	Radius = 0.26/ 25	2.45/5.8	10.9 (Meas)	−5.1 (2.45 GHz) 3.3 (5.8 GHz) (Mea)	Yes	16 (2.45 GHz) 54 (5.8 GHz) (Sim)
[32]	0.63 × 0.57/ 60 × 55	2.45	12.88 (Sim)	Not reported	Yes	74–90 (Sim) Over the entire operating bandwidth region
[22]	0.84 × 0.42/ 50 × 25	2.45	~34.39 (Sim)	5.2 (Sim)	Yes	72.1 (Sim)
[33]	0.77 × 0.58/ 80 × 60	2.45	12.8 (Sim)	6.07 (Sim)	Yes	59 (Sim)
[34]	0.57 × 0.49/ 53.6 × 45.8 (of the patch)	2.45	6.1 (Meas)	7.8 (Meas)	Yes	90 (Meas)
[35]	0.51 × 0.91/ 50 × 19 (PIFA antenna)	2.45 (Sim) 2.63 (Meas)	28 (Sim) 31 (Meas)	1.78 (Sim) 1.2 (Meas)	Yes	N/A
This work	0.35 × 0.17/ 50 × 25	1.87/2.45 (Sim) 1.8/2.38 (Meas)	49.04 (Sim) 40.16 (Meas)	2.5 (1.87 GHz) 3 (2.45 GHz) (Sim)	Yes	93 (1.87 GHz) 93 (2.45 GHz) (Sim)

λg = lower operating wavelength, Sim = simulated, Meas = measured.
